# Decoding miRNA-Mediated Immunoregulation in SARS-CoV-2, HBV, HIV, and HSV Infections

**DOI:** 10.1038/s41435-026-00376-4

**Published:** 2026-01-20

**Authors:** Suleyman Arziman, Sevim Aydemir, Vildan Bozok

**Affiliations:** 1 Department of Biotechnology, Faculty of Bioengineering, Ege University, Izmir, Türkiye; 2 Department of Medical Biology, Faculty of Medicine, Ege University, Izmir, Türkiye

**Keywords:** Infection, Antigen-presenting cells

## Abstract

Eukaryotic cells regulate gene expression through multiple checkpoints, including post-transcriptional mechanisms mediated by microRNAs (miRNAs). These small non-coding RNAs inhibit translation by binding to target mRNAs, often within a complex regulatory network involving other RNA species such as circular RNAs and long non-coding RNAs. miRNAs are now recognised as central players in the pathogenesis, immune modulation, and progression of infectious diseases. In this review, we thoroughly examine studies published over the past five years, focusing on miRNAs involved in immune regulation during four major viral infections: severe acute respiratory syndrome coronavirus 2, hepatitis B virus, human immunodeficiency virus, and herpes simplex virus. Our analysis centres on the core signalling pathways most frequently targeted by miRNAs: NF-κB, MAPK, JAK-STAT, TGF-β/Smad, and pattern-recognition receptor-associated cascades. Among the miRNAs most prominently implicated are miR-21, miR-146a, miR-150, and miR-155. These miRNAs modulate key signalling pathways, thereby influencing macrophage polarisation, T- and natural killer cell activity, antigen presentation, and inflammatory cytokine production. In addition, virus-encoded miRNAs and ceRNA or extracellular vesicle-mediated interactions are discussed where mechanistically validated, illustrating virus-specific regulatory layers. Collectively, this integrative synthesis underscores the pivotal roles of miRNAs in orchestrating antiviral immunity and highlights their potential as biomarkers and therapeutic targets in viral infections. A better understanding of miRNA-mediated immunoregulation may pave the way for precision interventions aimed at improving immune control and patient outcomes.

## Introduction

The innate immune system provides the first line of defence against viral infections. Dendritic cells (DCs) and macrophages are among the earliest sentinels to detect invading pathogens, initiating cytokine production and antigen presentation that bridge innate and adaptive immunity. Pattern-recognition receptors (PRRs) -including Toll-like receptors (TLRs), RIG-I-like receptors- sense viral nucleic acids and trigger the production of interferons (IFNs) and pro-inflammatory cytokines [1]. Natural killer (NK) cells are activated by the downregulation of major histocompatibility complex class I (MHC-I) molecules and the expression of stress-induced ligands on infected cells.

IFNs, in turn, induce a broad spectrum of interferon-stimulated genes (ISGs) such as OAS1, MxA, and ISG15, which collectively restrict viral replication, enhance antigen presentation, and modulate the subsequent adaptive immune response, in which T- and B- lymphocytes recognise specific viral antigens. Activated T-cells directly target infected cells, while activated B-cells secrete antibodies that neutralise the viruses by preventing their binding to and entry into host cells. This coordinated innate-adaptive transition is essential for effective viral clearance. However, in severe cases, dysregulation of this sequence such as delayed IFN production or hyperactivated cytokine release can lead to immune pathology rather than protection [2]. This highlights the need to better understand regulatory checkpoints at each immune phase.

Unlike phosphorylation-based protein signalling, miRNAs provide an additional, fine-tuned layer of post-transcriptional control that can either amplify innate responses -for example, by enhancing IFN and pro-inflammatory cytokine expression- or, conversely, facilitate immune evasion and viral persistence [3]. Certain miRNAs are even co-opted by viruses to repress host restriction factors, supporting replication or latency [4].

Dysregulation of miRNA expression profiles is increasingly associated with disease progression in both chronic and acute infections. In defining the scope of this review, we comprehensively surveyed recent literature on miRNA regulation in viral infections. Among the numerous viral pathogens investigated, severe acute respiratory syndrome coronavirus 2 (SARS-CoV-2), hepatitis B virus (HBV), human immunodeficiency virus (HIV), and herpes simplex virus (HSV) consistently emerged as the most extensively studied and clinically relevant over the past five years. Therefore, we focused on four major viral pathogens to delineate conserved and virus-specific miRNA-mediated strategies that influence disease trajectory and immune outcome. Notably, several viruses also encode their own miRNAs, which may interact with or antagonise host miRNA networks, adding another layer of immune regulation. Many of these miRNAs converge on shared signalling pathways such as NF-κB, MAPK, JAK-STAT, and TGF-β/Smad enabling comparisons across viral pathogens. By centring on these four infections and the pathways they engage, this review integrates the most recent and robust evidence to provide a coherent framework for understanding miRNA-mediated immune regulation across diverse viral contexts.

## SARS-CoV-2

SARS-CoV-2 is a single-stranded, positive-sense RNA virus belonging to the coronavirus family. The virus’s spike protein binds to angiotensin-converting enzyme 2 (ACE2) receptors found on human cells, enabling viral entry [5, 6]. The respiratory tract, which has a high concentration of ACE2 receptors, is the primary target, making the infection potentially deadly by causing severe respiratory illness [7, 8]. The affinity of SARS-CoV-2 for ACE2-expressing cells not only defines its tissue tropism but also amplifies its capacity to trigger widespread inflammation and systemic complications. The early interaction between the spike protein and ACE2 remains a central focus in therapeutic design, especially in strategies aiming to block viral entry or downregulate ACE2 expression.

### miRNA-Mediated Regulation of SARS-CoV-2 Entry

**Fig. 1:**
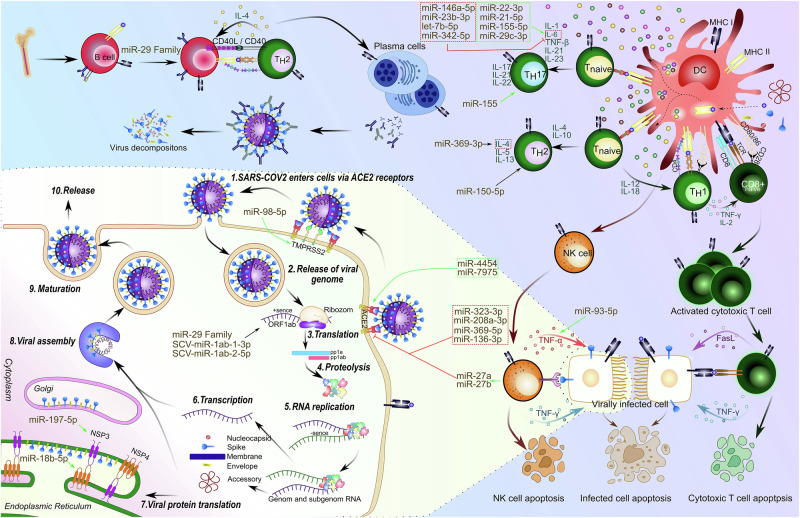
Life cycle of SARS-CoV-2 and regulatory effects of miRNAs on both viral replication and immune response. **1, 2** SARS-CoV-2 infection is initiated by viral binding to the ACE2 receptor on the host cell surface. The host protease TMPRSS2 cleaves the S2 domain of the viral spike protein, facilitating membrane fusion and allowing the release of viral RNA into the cytoplasm. **3, 4** The positive-sense viral RNA is translated by host ribosomes, particularly the ORF1ab region, into polyproteins, which are subsequently cleaved into functional viral proteins. **5–7** RNA replication proceeds through both full-length and subgenomic RNA synthesis. Structural (S, M, E, N) and accessory proteins are produced and processed through the endoplasmic reticulum and Golgi. **8–10** Mature virions are assembled and released via vesicular transport to initiate further rounds of infection. TMPRSS2 expression is positively regulated by miR-98-5p, enhancing viral entry. ACE2 expression is modulated by exosomal miRNAs released from immune cells: miR-27a/b (primarily from NK cells), along with miR-323-3p, miR-208a-3p, miR-369-5p, and miR-136-3p (from macrophages and other immune cells), act to suppress ACE2 expression, limiting viral entry. Conversely, miR-4454 and miR-7975 upregulate ACE2, potentially increasing susceptibility to infection. The endoplasmic reticulum-associated protein NSP3 is targeted by miR-197-5p, while NSP4 is regulated by miR-18b-5p, potentially affecting replication complex formation and stability. During RNA synthesis and transcription, both the miR-29 family and SCV-derived miRNAs (SCV-miR-1ab-1-3p and SCV-miR-1ab-2-5p) are actively involved in controlling viral gene expression. Antigen presentation by DCs activates naïve CD4^+^ T-cells, which differentiate into TH1, TH2, and TH17 subsets under the regulation of miR-155, miR-150-5p, and miR-369-3p, respectively. Notably, IL-4 production by TH2 cells is regulated by miR-369-3p, influencing TH2 polarisation and function. Activated TH2 cells stimulate B-cell activation and plasma cell differentiation, with antibody production further modulated by the miR-29 family. Among NK cell-derived factors, TNF-α expression is upregulated by miR-93-5p, contributing to apoptosis of infected targets. Alongside FasL-mediated pathways, TNF-α signalling supports effective immune clearance; however, excessive activation may also induce apoptosis in immune effector cells, contributing to immune dysregulation. Moreover, IL-6 expression is tightly regulated by a distinct set of miRNAs. Negative regulators include miR-146a-5p, miR-23b-5p, let-7b-5p, and miR-342-5p, which suppress IL-6 transcription. In contrast, miR-22-3p, miR-21-5p, miR-155-5p, and miR-29c-3p enhance IL-6 production, collectively modulating the intensity and duration of inflammatory responses. Created with İnkscape.

The ACE2 receptor is subject to intricate miRNA-mediated regulation (Fig. 1). For example, miR-1246 targets and regulates ACE2 expression [9], miR-369-5p and miR-136-3p downregulate ACE2 under the influence of TGF-β1 [10]. miR-27a and miR-27b, delivered via NK cell-derived extracellular vesicles (NK-EVs), also target ACE2 to prevent viral entry [11]. Additionally, ACE2-expressing exosomes exhibit higher levels of let-7g-5p and miR-4454 + miR-7975, and lower levels of miR-208a-3p and miR-323-3p compared to ACE2-negative exosomes [12].

Although several host-derived miRNAs have been reported to regulate ACE2 expression – such as miR-27a/b (from NK cells), miR-136-3p and miR-369-5p (under TGF-β1 influence), and exosomal miR-323-3p, miR-208a-3p, miR-4454, and miR-7975 to date, no peer-reviewed experimental evidence supports that a viral-encoded miRNA directly modulates ACE2 in mammalian cells. Current data are limited to host miRNAs, whereas viral miRNAs described in SARS-CoV-2 primarily target type I IFN pathways (e.g., STAT1/2) rather than ACE2 [4]. This remains an important open question, and future studies should explore whether viral miRNAs or miRNA-like molecules may directly influence ACE2 or its regulatory networks, which would provide valuable mechanistic insight into coronavirus pathogenesis.

Beyond ACE2, several miRNAs regulate additional receptors involved in SARS-CoV-2 entry, including NRP1 and TMPRSS2. miR-98-5p targets TMPRSS2 in endothelial cells, and downregulation of this protease may limit viral entry and fusion, suggesting a potential protective role in SARS-CoV-2 infection [5]. These findings underscore a shared regulatory axis where miRNAs converge on SARS-CoV-2 entry receptors to modulate infection severity.

### SARS-CoV-2 Viral miRNAs and Host-Virus Interplay in Interferon Signalling

Viral miRNAs such as SCV2-miR-OR1ab-1/2 have been shown to target type I IFN pathway by suppressing STAT1 and STAT2, thereby suppressing host antiviral responses and facilitating immune evasion [13, 14]. SARS-CoV-2-encoded proteins also directly interact with human RNA-binding proteins (RBPs) expressed in immune cells, potentially influencing host RNA regulatory networks. A computational approach using FIMO was employed to identify human miRNA binding sites across the SARS-CoV-2 genome, revealing 22 miRNAs with predicted interactions at various loci [15].

The viral genome’s interaction with host miRNAs, such as miR-197-5p and miR-18b-5p, highlights evolutionary strategies to evade miRNA-mediated inhibition. These miRNAs target regions within NSP3 and NSP4, with mutations in these viral genes facilitating immune escape [16]. miR-29 family have a total of 11 binding sites to SARS-CoV-2 genome sequence mainly targeting ORF, spike (S) and nucleocapsid (N) regions [17]. miR-1207-5p targets S region, deregulates CSF-1 to enhance inflammatory responses [18]. This interplay between host and viral miRNAs exemplifies a competitive regulatory dynamic driven by co-evolution. Understanding this molecular tug-of-war may prove critical for the development of RNA-based antiviral strategies.

### miRNA-Mediated regulation of inflammatory responses in SARS-CoV-2 Infection

miRNAs involved in the SARS-CoV-2 infection are summarised in Supplementary Table 1 and Fig. 1 Among these, miR-155 emerges as a pivotal regulator influencing multiple immune and inflammatory responses. Studies have shown that miR-155 targets key immune pathways, including cytokine signalling and the NF-κB, which regulates the expression of pro-inflammatory cytokines like TNF-α and IL-6, influencing immune cell activity and inflammatory responses [19, 20]. Specifically, upregulation of miR-155 in SARS-CoV-2 infection correlates with downregulation of SOCS-1, promoting Th17 differentiation and production of pro-inflammatory cytokines such as IL-17 and IL-21 [21]. This dysregulation is further linked to decreased regulatory T-cell (Treg) activity, as seen in reduced FoxP3 expression [21]. Together with miR-200c-3p, miR-155-5p serum levels are discriminated between mild and moderate cases [22]. These findings collectively suggest that miR-155 serves as an important regulator within a broader network of miRNAs and transcriptional factors that coordinate inflammation and immune polarisation.

Likewise, miR-146a, another immune modulating miRNA, exhibits dual regulatory effects [23]. miR-146a-5p acts in an anti-inflammatory manner by inhibiting components of the NF-κB pathway, such as IRAK1 and TRAF6, during macrophage polarisation and cytokine stimulation, leading to a decrease in pro-inflammatory cytokines like IL-6 and IL-8 [23]. Conversely, miR-146a-3p appears to promote inflammation, thus having regulatory functions that vary based on cell type [23]. miR-146a-5p was also related to IFI44 expression which showed positive correlation between immune cell infiltration in activated dendritic cells [24]. Together, these findings suggest that miR-146a-mediated signalling serves as a crucial node in balancing inflammatory responses. This cell-type dependent effect of miR-146a and its isoforms reveals a sophisticated level of immune tuning, where the same miRNA family may exert both suppressive and stimulatory roles depending on context. Therapeutic modulation of miR-146a must therefore consider cellular heterogeneity and disease stage.

Moreover, miR-369-3p has been linked to inflammation as well as immune homeostasis. Its low levels have been associated with severe COVID-19 cases requiring mechanical ventilation [25]. miR-369-3p regulates several immune pathways including T-cell receptor and co-stimulatory signalling; IL-4, Notch, and TGF-β pathways, underscoring its role in limiting immune dysregulation and fibrosis [25]. As previously discussed, miR-369-3p also downregulates ACE2 expression, thereby potentially limiting viral entry [7, 10]. Collectively, the involvement of miR-369-3p in antiviral defence, anti-fibrotic activity, and immune regulation highlights its promise as both a therapeutic target and a biomarker for clinical deterioration.

Additionally, downregulation of miR-150-5p during early SARS-CoV-2 infection coincides with increased STAT1 expression, promoting inflammatory responses, while its later normalisation aids in resolving immune perturbations [26]. This temporal regulation underscores the dynamic interplay of miRNA-mediated immune responses. Such time-sensitive miRNA expression patterns provide insights into the staged nature of COVID-19 immunity. miR-150-5p may have potential as a diagnostic biomarker and as a candidate for temporally controlled immune modulation. miR-16–2-3p and miR-618 are other miRNAs that reported as diagnostic and prognostic effects in the regulation of immune response in SARS-Cov-2 [27]. A multi-omics study of 259 unvaccinated COVID-19 patients linked genotyping, miRNA/mRNA expression, and clinical data to disease severity. Among 632 miRNAs analysed, 97 were associated with blood traits predictive of intensive care unit (ICU) admission [28]. Notably, miR-143-3p regulated neutrophil count via BCL2, and 168 cis-miRNA eQTLs were identified, 57 of which correlated with ICU risk or blood phenotypes [28].

### Non-Coding RNA Networks and Extracellular Vesicle-Mediated miRNA regulation in SARS-CoV-2 Infection

Competitive endogenous RNA (ceRNA) networks involving circRNAs, miRNAs, and mRNAs orchestrate immune responses by mediating interactions between viral and host RNAs. In COVID-19 patients, a ceRNA network involving miR-20a-5p, miR-29b-2-5p, miR-142-5p, miR-505-5p, and miR-6501-5p may contribute to pathogenesis via NF-κB signalling [29]. Additionally, SARS-CoV-2-derived circ_3205 interacts with miR-298, upregulating KCNMB4 and PRKCE mRNAs [30], while the LINC01002-miR-4324-FRMD8 axis mediates N protein-driven immune evasion by suppressing IFN-β induction [31].

Activated immune cells release extracellular vehicles (EVs) containing miRNAs that enter the circulation and can be detected as potential biomarkers. Notably, reduced circulating levels of miR-150-5p and miR-93-5p have been linked to a higher risk of COVID-19-related mortality in cancer patients [32]. Serum miRNA signatures, characterised by elevated miR-21-5p and miR-22-3p targeting antiviral response genes, and reduced miR-224-5p and miR-155-5p targeting pro-inflammatory factors, effectively discriminated severe from mild/moderate cases [33].

The therapeutic potential of EVs derived from various immune cells has been investigated, and their antiviral efficacy associated with their miRNA cargo. For instance, NK-EVs, carrying anti-viral miRNA cargo (miR-27a, miR-27b, miR-369-3p, miR-491-5p) reduced viral RNA and pro-inflammatory cytokines (TNF-α, IL-8) in SARS-CoV-2 infected cells and mice [11]. Peritoneal M2-derived extracellular vesicles (M2-EVs) carrying functional cargos (proteins and miRNAs) effectively suppress pro-inflammatory pathways, including NF-κB and JAK-STAT signalling, as demonstrated by reduced TNF-α and IL-6 levels [34]. MSC-derived extracellular vesicles (MSC-EVs) attenuated COVID-19 pathology by delivering miRNA cargo -including miR-7, miR-18a, miR-29b, miR-124, miR-145, miR-146a, miR-155- into lung tissue, thereby suppressing the JAK-STAT signalling pathway and reducing cytokine storm and coagulopathy [35]. EVs can carry endocannabinoids in COVID-19 along with miRNAs to regulate immunologically active molecules such as VEGFA, GNAI2, IGF1, BDNF, IGF1R and CREB1 [36]. These findings provide a mechanistic rationale for exploring EV-based delivery systems in clinical settings. By harnessing miRNAs that restore immune homeostasis, such approaches may offer targeted interventions without broadly suppressing host immunity.

Finally, miRNA regulation can be influenced by epigenetic modifications [37], and may exhibit regional variability [38]. Using Network Analyst, a transcription factor–miRNA–hub gene regulatory network (56 nodes, 56 edges) revealed 28 miRNAs and 26 transcription factors interacting with TOP2A and CEP55, with ATF1 and miR-144 as shared regulators. A parallel protein–drug interaction analysis predicted 33 candidate drugs targeting TOP2A [39].

Collectively, these findings demonstrate that host- and virus-derived miRNAs operate within an interconnected regulatory network rather than as isolated factors. They converge on central signalling axes —ACE2/TMPRSS2-mediated viral entry, interferon, and NF-κB/JAK-STAT pathways— forming a coordinated framework that shapes the outcome of SARS-CoV-2 infection. Pro-inflammatory miRNAs such as miR-155 and miR-146a act as reciprocal regulators balancing cytokine production and antiviral activity, whereas anti-inflammatory or reparative miRNAs including miR-21, miR-150-5p, and miR-369-3p promote resolution of inflammation and tissue recovery. Viral miRNAs further modulate host responses, contributing to immune evasion through ceRNA interactions and extracellular vesicle pathways. Together, these multilayered miRNA circuits define the fine balance between protective immunity and pathological inflammation in COVID-19, offering a mechanistic framework that links molecular regulation to clinical outcomes and highlighting miRNAs as promising biomarkers and therapeutic targets for disease management.

## Hepatitis B virus

Hepatitis B virus is a persistent viral pathogen that establishes chronic infection by evading host immune responses through immune tolerance, T-cell exhaustion, and viral mutation. Effective immune responses typically result in acute, self-limiting hepatitis; however, HBV can escape host immune clearance through the depletion of CD8^+^ T-cells, leading to chronic hepatitis, cirrhosis, and hepatocellular carcinoma. This progression from acute to chronic infection underlines the importance of immune exhaustion, especially of CD8^+^ T-cells, as a central determinant of viral persistence [40]. The depletion of these effector cells not only signifies immune failure but also indicates a potential therapeutic window where reinvigoration of T-cell function could alter disease trajectory.

### miRNA-Mediated modulation of innate and adaptive immunity in HBV infection

As shown in Supplementary Table 2, HBV infection and associated immune responses are regulated through complex miRNA-driven mechanisms. Analysis of differentially expressed miRNAs in HBV-infected patients has revealed expression signatures underlying immune regulation and pathogenesis [41–43]. Distinct miRNA expression patterns were identified in DCs across different stages of infection, revealing 19 miRNAs interacting with 12 target genes involved in antigen processing and immune pathways [44]. Notably, miR-2278, miR-615-3p, and miR-3681-3p were downregulated in the immune-active group, leading to upregulation of immunoregulatory genes such as CANX, HSPA8, HSPA1B, and HSP90AB1, which are crucial for MHC-I assembly and DC maturation [44]. These findings suggest that dysregulated miRNA–mRNA networks in DCs contribute to impaired antigen presentation and viral persistence in HBV pathogenesis.

Another important miRNA, miR-128-3p, binds to the C allele variant (rs2844512) of LINC01149, leading to increased expression of MICA, a key activator of NK-cell cytotoxicity [45]. This allele-specific regulation highlights how host genetic variation can influence the strength of antiviral responses. Elevated MICA expression may enhance early innate immunity and improve control of HBV replication. Virus-induced miR-30e enhances innate immune responses by post-transcriptionally suppressing negative regulators of PRR signalling, thereby limiting viral replication [46]. Elevated miR-30e levels in therapy-naïve HBV patients further support its potential as a biomarker of antiviral immune activation.

miRNAs also shape immune responses during fibrogenesis in HBV-related liver cirrhosis (HBV-LC). Network analysis of 158 differentially expressed miRNAs and 442 mRNAs identified a core module involving miR-20a-5p and miR-340-5p, which regulate genes associated with inflammation, wound healing, and leukocyte migration [47]. Functional assays confirmed their role in macrophage polarisation and hepatic stellate-cell activation, linking miRNA-mediated immune regulation to fibrotic progression and highlighting potential targets for prognostic or therapeutic intervention.

Further, co-regulatory miRNA–mRNA networks have been described in acute-on-chronic liver failure (ACLF) due to HBV [48]. miR-6840-3p and miR-6861-3p were upregulated in patients with poor outcomes, while miR-93, miR-106b, miR-210, and miR-374b were associated with prognosis, and miR-125b downregulation correlated with worse outcomes [49]. miR-93 targets GRB2, a gene influencing immune-cell infiltration in hepatocellular carcinoma, affecting CD4⁺ T-cells, macrophages, and neutrophils [49]. Additionally, in chronic HBV infection, miR-330-3p downregulation coupled with lnc-AIFM2-1 upregulation promotes CD244 expression on CD8⁺ T-cells, contributing to immune exhaustion and viral persistence [50].

### Viral and host miRNAs targeting signalling pathways in HBV infection

Among viral miRNAs, HBV-miR-3 plays a pivotal role by suppressing SOCS5, activating the JAK-STAT pathway, and enhancing antiviral gene expression (OAS1, MX1, IFIT2, IFIT3) in response to IFN-α [51]. HBV-miR-3 also promotes M1 macrophage polarisation via STAT1 phosphorylation and increases IL-6 production [51]. This dual function -boosting antiviral defence while promoting inflammation- reflects a delicate balance between viral control and immunopathology, underscoring the need to weigh both protective and potentially harmful consequences of immune activation in HBV infection.

The MAPK pathway is another critical hub of miRNA-mediated regulation. miR-212-3p is upregulated in HBeAg-stimulated macrophages via the ERK/CREB signalling axis and acts as a negative regulator of inflammation by targeting MAPK1, thereby suppressing cytokine production [52]. In contrast, miR-939 exerts a proinflammatory effect by enhancing cytokine production through the MAPK-p38-IL8 pathway in monocytes [53]. miR-939, delivered via subviral particles, increases IL-8 through p38-MAPK activation, correlating with both inflammation and fibrosis stage in HBV-associated liver disease. Likewise, miR-138-5p targets A3B, a cytidine deaminase with antiviral activity against HBV [54]. While NF-κB signalling is essential for A3B induction, miR-138-5p promotes A3B mRNA decay, thereby limiting its antiviral effects [54].

From a translational perspective, RNA interference (RNAi) has emerged as a promising therapeutic avenue. A trimeric artificial miRNA (amiRNA135) targeting conserved HBV genome regions effectively inhibited viral replication across genotypes, including resistant strains [55]. Delivered via AAV8 vectors, it reduced HBsAg and HBeAg levels in vivo in a time- and dose-dependent manner, with sustained effects for up to 15 months. These results highlight amiRNA135 as a promising RNAi-based strategy for chronic HBV treatment [55].

Together, these findings indicate that HBV infection engages a complex miRNA network that fine-tunes antiviral immunity, inflammation, and fibrogenesis through convergent regulation of the JAK-STAT, NF-κB, and MAPK pathways. Host miRNAs such as miR-30e, miR-128-3p, and miR-340-5p orchestrate immune activation, DC function, and fibrotic signalling, while virus-encoded miRNAs like HBV-miR-3 exploit the same pathways to maintain persistence and immune evasion. This interplay between host and viral miRNAs defines the balance between immune control and immunopathology, ultimately shaping clinical outcomes. Integrating these insights reveals a unified regulatory framework linking immune activation, metabolic homeostasis, and liver pathology. Future research employing multi-omics approaches and targeted RNA-based therapies will be instrumental in translating these mechanistic discoveries into clinical interventions for chronic HBV-associated disease.

## Human immunodeficiency virus

Human Immunodeficiency Virus continues to represent a major global health challenge due to its progressive impairment of the immune system. Although antiretroviral therapy (ART) has transformed HIV into a manageable chronic condition, it requires lifelong adherence, remains costly, and may be associated with toxic side effects. These limitations have driven efforts to identify strategies enabling ART-free remission in HIV management [56, 57]. A particularly informative model in this context is that of elite controllers (ECs) -individuals who maintain undetectable viral loads without ART [58]. In these individuals, robust cytotoxic CD8⁺ T-cell responses play a central role in viral suppression, underscoring the critical importance of cellular immunity in controlling HIV-1 replication.

HIV-1 reshapes host immunity through miRNA-mediated networks that influence viral entry, replication, pathogenesis, T-cell function, and clinical trajectories. Below, we organise HIV-associated miRNAs into functional themes (summarised in Supplementary Table 3).

### miRNA-Mediated regulation of HIV-1 entry and replication

HIV-1 entry begins with envelope (Env) engagement of CD4 and co-receptors (CCR5, CXCR4) following adhesion to host membranes. Several host miRNAs modulate this step or early post-entry events. miR-103 reduces CCR5 expression in primary CD4⁺ T-cells through a p53-dependent mechanism, limiting the establishment of latent HIV-1 reservoirs in vivo [59]. miR-191-5p inhibits HIV-1 replication by targeting CCR1 and the nuclear import factor NUP50, thereby impairing entry/replication [60]. miR-29a-3p suppresses replication by targeting the viral *nef* gene, a key regulator of immune evasion and infectivity. Importantly, serum levels of miR-29a-3p are negatively correlated with viral load in clinical samples [61]. Conversely, downregulation of miR-125b in infected cells increases CPSF6, facilitating nuclear import and integration [62].

HIV-1 also reprogrammes host miRNAs to its advantage. The viral protein Vpr induces miR-210-5p expression via NF-κB (p50) phosphorylation, promoting cell cycle arrest and facilitating viral persistence [63]. Tat upregulates miR-505, which suppresses the SIRT3/antioxidant axis and drives mitochondrial dysfunction and senescence [64]. Together, these findings illustrate a dynamic interplay in which host miRNAs restrict HIV-1 entry and early replication, while viral proteins subvert these defences by modulating miRNA expression to establish infection and latency.

### miRNA-Mediated regulation of T-cell function in HIV-1 Infection

miRNAs shape T-cell phenotype, effector function, and immune recovery. In CD8⁺ T-cells, e*x vivo* blockades of miR-10a-5p in HIV-1 specific CD8⁺ T-cells from both elite controllers and ART-treated individuals significantly enhanced antiviral activity [65]. miR-10a-5p targets several genes involved in host-virus interactions and immune regulation. Its inhibition led to increased expression of key effector molecules, including granzyme B and IFN-γ, suggesting that targeting miR-10a-5p could be a promising strategy to reinvigorate cytotoxic T-cell function in HIV infection.

Elevated plasma ATP in the HIV-positive individuals can upregulate miR-30b/c/e expression and downregulate CD73 on CD8⁺ T-cells [66]. CD73 is essential for the production of immunosuppressive adenosine. Its loss was associated with reduced secretion of TNF-α and IFN-γ and impaired cytolytic function, revealing a miRNA-mediated mechanism contributing to T-cell exhaustion.

Beyond effector function, miRNAs also influence immune recovery following ART. A five-miRNA plasma signature (miR-16-5p, miR-138-5p, miR-323-3p, miR-580, and miR-627) associates with poor CD4⁺ T-cell recovery in ART-treated individuals, suggesting that these circulating miRNAs could serve as early biomarkers of immunological non-response [67]. miR-16-5p directly inhibits CD4⁺ T-cell proliferation by modulating intracellular calcium flux, highlighting a mechanistic link between miRNA expression and impaired immune reconstitution [67]. Finally, secretion of miR-15a and miR-24 by HIV-1 infected CD4⁺ T-cells suppresses CD34⁺ progenitor differentiation, potentially restricting T-cell lineage replenishment at the source [68].

### Circulating miRNAs as prognostic and diagnostic biomarkers

Distinct circulating and intracellular miRNA patterns correlate with disease states and treatment timing [56, 58, 69, 70]. For instance, miR-27b, miR-29a, miR-150, and miR-221 differ between long-term non-progressors and ART-naïve individuals, correlating with viral load and CD4⁺ counts [69]. Delayed ART initiation is associated with PBMC miRNA signatures indicating heightened immune activation; early starters show upregulation of miR-155-5p/miR-1248 and downregulation of miR-501-3p, miR-548d-5p, miR-18a-3p, miR-296-5p, correlating with immunological markers (CD4⁺ counts; CD69 on CD4⁺ T-cells; HLA-DR on CD16^high^ NK subsets) [58]. In acute HIV-1 infection, miR-122-5p rises with innate activation and early angiogenic signals [71]. As for ART responses, let-7d-5p is reduced in immunological non-responders after prolonged suppression, suggesting utility for monitoring immune recovery [72]. In broader settings, miR-150 predicts survival in Pneumocystis pneumonia, while miR-320a-3p is elevated in HIV/TB co-infection and inversely correlates with FKBP5 expression [73, 74]. Beyond cardiovascular risk (plaque-linked miRNAs) [75], plasma miR-127-3p and miR-485-5p associate with domain-specific cognitive impairment [76], and miR-192 is altered in immune-recovery uveitis [77].

Integrated miRNA–mRNA profiling in ART-treated cohorts reveals cascaded regulatory relationships affecting inflammatory and immune pathways, underscoring the value of paired omics for tracking immune recovery and prognosis [78]. Collectively, these data support miRNAs as stable, non-invasive biomarkers spanning disease stage, treatment timing, and co-morbidities.

### Extracellular vesicle-associated miRNAs in HIV-1 pathogenesis

Extracellular vesicles serve as vehicles for miRNAs that influence HIV-1 pathogenesis through intercellular communication [79–81]. In ART treated individuals, circulating EVs are more abundant and enriched for specific miRNAs (e.g., miR-21-5p, miR-27b-3p, miR-146a-5p, and miR-423-5p) correlating with oxidative stress and immune dysregulation, and offering biomarker potential [82]. Plasma extracellular miRNAs (exmiRNAs) distribute across both lipid-based EVs and non-lipid extracellular condensates (ECs); with approximately 30% of exmiRNAs is associated with ECs [83]. In SIV-infected rhesus macaques, miR-128-3p is persistently downregulated in EVs, but not in ECs, suggesting a conserved progression marker [83].

In the central nervous system, astrocyte-derived EVs, induced by Tat, are enriched for miR-7, which is transferred to neurons where it downregulates neuroligin-2 and disrupts synaptic structure. Notably, this neurotoxic effect is reversible by PDGF-CC treatment [80]. In cerebrospinal fluid-derived EVs from cerebral toxoplasmosis/HIV co-infection, miR-155-5p and miR-21-5p are upregulated [79]. Basal ganglia derived EVs (BG-EVs) displayed downregulation of 11 miRNAs, whereas treatment with delta-9-tetrahydrocannabinol (THC) upregulated 37 miRNAs linked to TLR/TRK signalling and cell-death pathways [81]. Overall, EV-miRNAs act as biomarkers and mediators of neuroinflammation, immune signalling, and co-infection dynamics in HIV-1 pathogenesis.

### Drug-vaccine associated miRNA signatures and therapeutic potential

HIV-1 infection and exposure to various pharmacological agents significantly influence miRNA expression, thereby shaping viral susceptibility, immune modulation, and comorbidity outcomes. For example, HIV-1 induced downregulation of miR-26a enhances CD59 packaging, reducing complement-mediated lysis; restoration of miR-26a reverses this resistance and restores complement sensitivity [84]. Methadone suppresses key viral restriction factors in macrophages, thereby facilitating HIV-1 replication through miRNA-dependent mechanisms [85]. Methamphetamine enhances HIV-1 replication in CD4⁺ T-cells via an IL-1β–driven auto-regulatory loop [86].

Drug-modulated miRNAs also offer therapeutic angles. Zoledronic acid, alleviates HIV-associated bone loss by upregulating miR-302, miR-101, and miR-145 (targeting PRKACB, RANKL and SMAD3), thereby limiting osteoclast genesis [87]. Opiates alter miRNA content in EVs, potentially reshaping immune communication and HIV pathophysiology [88]. In the central nervous system, co-exposures to Tat and cocaine disrupt astrocytic energetics through the LINC01133–miR-4726-5p–NDUFA9 axis, linking epigenetic control to mitochondrial dysfunction [89].

In HIV-positive individuals vaccinated with yellow fever virus, distinct cytokine, receptor, and miRNA signatures indicate altered antiviral signalling in immunocompromised hosts [90]. Similarly, in a dendritic cell-based therapeutic HIV-1 vaccine trial, early activation of inflammatory and complement pathways and downregulation of miR‑223‑3p, miR‑1183, and miR‑8063 predicted better viral control following analytical treatment interruption, implicating miRNA tuning in vaccine efficacy [91]. In non-human primates, a prophylactic DNA/ALVAC/gp120/alum vaccine demonstrated that both adaptive and innate responses contribute to protection [92]. Notably, miR-139-5p was upregulated and inversely correlated with suggesting enhancement of anti-inflammatory cAMP signalling [92].

HIV-1 exacerbates metabolic and organ-specific comorbidities through miRNA-mediated mechanisms. HIV-1 induces miR-33b-5p, which targets ABCA1, impairs cholesterol efflux and promotes lipid accumulation in macrophages; notably these effects is reversible upon inhibition of miR-33b-5p, offering a potential therapeutic avenue [93]. In the gastrointestinal tract, miR-1297 promotes barrier repair by negatively regulating PLCβ1, highlighting potential for managing HIV-associated intestinal dysfunction [94].

Across HIV-1 infection, miRNA networks act at multiple levels to shape disease course: (i) restricting or facilitating entry and replication; (ii) modulating CD8⁺ and CD4⁺ T-cell effector function, exhaustion, and immune reconstitution; (iii) serving as circulating biomarkers that reflect disease stage, ART timing, and co-morbidities; and (iv) mediating cell–cell communication via EVs, especially in neuroinflammation. Viral proteins (Tat, Vpr) further reprogramme host miRNAs to favour persistence, underscoring a tug-of-war between antiviral regulation and viral adaptation. Synthesising these observations, a unifying network emerges in which a limited set of signalling nodes (e.g., CCR5/entry machinery, NF-κB/IL-1β axes, mitochondrial stress pathways) are targeted by distinct miRNAs across contexts.

## Herpes simplex virus

Herpes simplex virus infections pose a significant challenge in both immunocompetent and immunocompromised individuals [95]. Patients who are deficient in NK cells frequently suffer from serious and recurrent HSV infections [96]. Such cases can be considered indicators of compromised antiviral responses, which may arise due to malformations in the number or functional capability of NK cells. However, some patients who experience severe HSV infections despite having normal numbers and cytotoxic activity of NK cells still lack a fully understood underlying cause [96]. This observation suggests that other regulatory mechanisms, such as the action of miRNAs, may play a critical role in antiviral defence.

### miRNA-Mediated regulation of NK-cell function in HSV infection

Specific miRNAs can suppress the harmful activities of NK cells, and it is understood that HSV has evolved strategies to overcome such immune control systems. For example, studies have found that patients with severe HSV infections present high levels of miR-27b, miR-199b, miR-369-3p, and miR-491-3p in NK cells (Supplementary Table 4). These miRNAs can regulate the expression of significant antiviral genes such as MAVS, TLR7, MAP2K3, and perforin; thereby influencing NK cell activation and effector function [96]. Such post-transcriptional silencing may explain susceptibility even when overt cellular defects are not evident, underscoring the utility of miRNA profiling for personalised risk assessment.

### Viral and host miRNAs targeting signalling pathways and latency

HSV-1 is not merely a passive target of host immunity but actively produces its own miRNAs to control the host environment. Latency-associated transcript (LAT)-encoded miRNAs (miR-H2, miR-H3, and miR-H4) target SMAD3 and SMAD4 and reshape TGF-β/Smad signalling, supporting survival and latency [95]. In a parallel study, miR-H8 deletion reduces viral titters in certain cell types while enhancing replication in neuron-like cells, highlighting the multifunctional, context-dependent roles of viral miRNAs [97].

Viral miRNAs also play a pivotal role in regulating the HSV-1 life cycle. Deletion of miR-H1 and miR-H6 has been shown to affect viral replication, LAT expression, and reactivation, suggesting that these miRNAs are critical in maintaining the delicate balance between latency and active infection through both Drosha-dependent and alternative mechanisms [98]. In glioma cells, the HSV-1 miRNA miR-H16 activates Notch signalling by directly targeting FIH-1, increasing levels of Notch ligands and downstream genes [99]. This process could improve the effectiveness of virotherapy used in cancer treatments. A closer examination of human trigeminal ganglia latently infected with HSV-1 reveals that while several HSV-1 miRNAs (miR-H2 to miR-H8) are detectable, miR-H4 is especially abundant [95]. Additionally, miR-H2 editing broadens its target range to include critical viral regulators like ICP0 and ICP4, suggesting that RNA editing may be a key regulatory node in the cycle between latency and reactivation [100].

The impact of HSV-1-derived miRNAs is not restricted to the peripheral nervous system. In neuroinflammatory and psychiatric diseases, cerebrospinal fluid exosomes consistently transport miRNAs like miR-H3-3p, miR-H6-3p, and miR-H27, whose levels correlate with neuroinflammatory markers and neuronal injury [101]. Furthermore, research on other disease conditions suggests crosstalk between regulatory mechanisms. Immune regulators and miRNAs such as miR-150-5p and miR-25-3p have been identified in networks among tuberculosis patients involving HSV-1 infection pathways, illustrating shared immune evasion strategies among various pathogens [102]. Environmental factors further complicate this landscape; toxins like Ar1260 have been shown to alter the expression of miR-155 and cytokines, affecting key inflammatory pathways such as NF-κB and TLR3 [103].

Insights from other herpesviruses provide further understanding. For instance, Kaposi’s sarcoma-associated herpesvirus encodes miR-K12-1-5p, which disrupts type I IFN signalling in cardiac cells, leading to myocarditis and dilated cardiomyopathy [104]. In severe myopia, miRNA dysregulation, including changes in miR-490-3p, miR-4423-3p, and miR-4485-3p, has been correlated with metabolic and inflammatory alterations, with some research implicating HSV-1 infection pathways, offering new therapeutic possibilities [105]. Additionally, bacterial virulence factors can interact with HSV transcripts, as Porphyromonas gingivalis gingipain has been shown to bind ICP4 and downregulate HSV miR-H6, thereby inducing latent reactivation and periodontal disease [106].

At the ocular level, HSV-1 infection of human corneal epithelial cells induces significant changes in circular RNAs and miRNAs like miR-338-3p, miR-181b-5p, and miR-635 [107]. These miRNAs act as molecular sponges to regulate gene expression, impairing antiviral defences and representing potential targets for treating diseases like herpes simplex keratitis [107]. The upregulation of the miR-183/96/182 cluster by fibroblasts during HSV-1 infection appears to mediate the host response through the FoxO family proteins, although their roles in directly inhibiting viral replication are limited [108]. The tissue-specific activity of these miRNAs in corneal epithelium and fibroblasts underscores their relevance in site-specific pathologies, especially where inflammation and tissue remodelling must be tightly regulated to preserve function, such as in the eye.

Beyond HSV infection itself, many other conditions are linked to miRNA dysregulation. In Parkinson’s disease, for instance, immune-related gene changes -many of which are involved in HSV-1 infection pathways- suggest that miRNAs may serve as both therapeutic targets and diagnostic markers [109]. In varicella-zoster virus-induced ischemic stroke, miR-29 and other miRNAs are involved in maintaining vascular integrity and regulating inflammation, with natural products like resveratrol identified as potential multitarget agents [110]. Additionally, tear samples from herpes epithelial keratitis patients exhibit distinctive miRNA profiles -such as elevated miR-29a-3p that could be used to distinguish between different ulcer types and detect underlying inflammatory processes [111].

In conclusion, HSV pathogenesis reflects a layered miRNA network in which host miRNAs modulate NK-cell antiviral functions and tissue-specific immunity, while viral miRNAs (particularly LAT-derived species) rewire TGF-β/Smad, Notch, and innate pathways to stabilise latency and shape reactivation. Beyond neuron-centric models, exosomal/vesicular miRNA transfer extends regulation to the central nervous system and ocular surface, and environmental or microbial co-factors can pivot these circuits toward inflammation or reactivation.

## Summary and conclusion

**Fig. 2:**
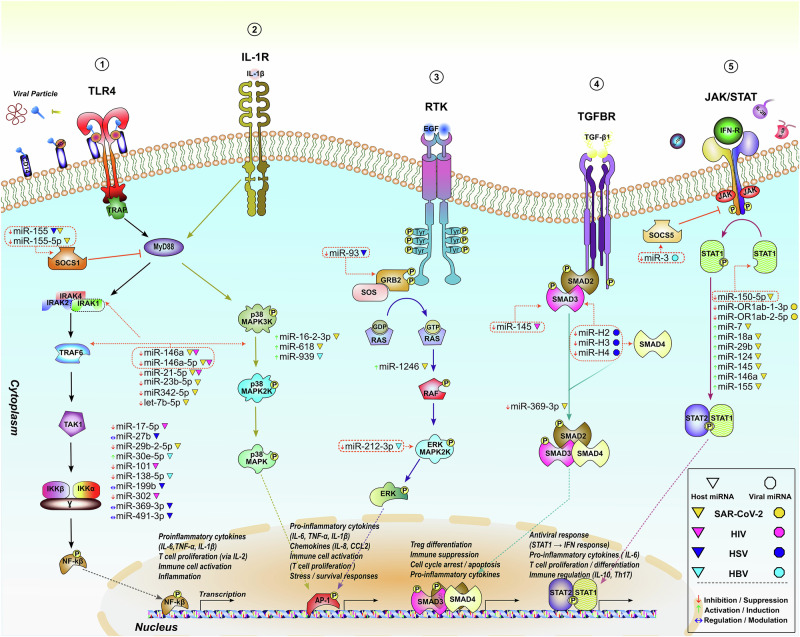
miRNA-mediated regulation of key signalling pathways. **1** | **PRR-induced NF-κB pathway:** Viral particles and proteins (e.g., SARS-CoV-2 Spike, HIV Tat/Nef, HBV X protein, HSV gB) as well as host cytokines (IL-1β, TNF-α) activate NF-κB signalling through PRRs such as TLR4. The NF-κB cascade is dynamically controlled by positive regulatory miRNAs (miR-155, miR-30e-5p), negative regulators (miR-21, miR-146a, miR-146a-5p), and context-dependent modulatory effects of HSV-associated host miRNAs. **2** | **IL-1R-mediated activation of the MAPK pathway:** IL-1R-induced signalling activates upstream MAP3Ks (e.g., TAK1), which engage the p38/MAPK pathway and culminate in activation of the AP-1 (Fos/Jun) transcription factor, driving transcription of pro-inflammatory genes. miR-146a targets IRAK1 and TRAF6, suppressing the IL-1R–MyD88–MAP3K axis. In contrast, miR-16-2-3p and miR-618 enhance p38 phosphorylation via MAP3K, promoting AP-1-mediated cytokine production, while miR-939 reinforces the pro-inflammatory output by upregulating IL-8 transcription. **3 | Receptor tyrosine kinase (RTK)–MAPK pathway:** RTKs (e.g., EGFR, FGFR, PDGFR) are activated by viral proteins (HIV gp120, HBV X, SARS-CoV-2 Spike) or host growth factors, transmitting signals through the RAS–RAF–MEK–ERK/JNK pathway. miR-93 targets the adaptor protein GRB2, thereby suppressing RTK–MAPK activation. Conversely, miR-1246 enhances ERK signalling and increases pro-inflammatory cytokine production. miR-212-3p directly represses MAPK1/ERK, limiting HBeAg-induced cytokine release in HBV. **4** | **TGF-β/SMAD pathway:** Activation of the TGF-β receptor complex leads to phosphorylation of SMAD2/3 and formation of a complex with SMAD4, which translocate to the nucleus to regulate immune suppression, cell-cycle control, and fibrotic processes. HSV-derived miR-H2, miR-H3, and miR-H4 target SMAD3/4, preventing complex formation. Host miR-145 also targets SMAD3, acting as a negative regulator. Additionally, miR-369-3p modulates TCR, Notch, IL-4, and TGF-β signalling during HSV infection, influencing T-cell polarisation. **5** | **JAK-STAT pathway:** Type I/II interferons and cytokines activate their receptors, leading to phosphorylation of JAK kinases and downstream STAT activation. The JAK-STAT pathway is dynamically regulated by both inhibitory miRNAs (miR-150-5p) and activating miRNAs (HBV-miR-3, miR-7, miR-18a), while SARS-CoV-2-encoded miRNAs act as potent suppressors that facilitate viral immune evasion. **Created with İnkscape**. *Host and viral miRNAs are indicated by triangles and octagonal, respectively, following the colour code for each infection: yellow – SARS-CoV-2, purple – HIV, blue – HSV, and cyan – HBV. The regulatory effects of individual miRNAs are depicted by arrows.

In this review, we thoroughly examined over one hundred publications to elucidate the immunoregulatory roles of miRNAs in four major viral infections—SARS-CoV-2, HBV, HIV, and HSV. Our aim was to define molecular miRNA patterns governing immune regulation and to identify the pathways through which these small RNAs influence viral replication, immune activation, and persistence. We centred our analysis on the most functionally validated miRNAs and the immune pathways they modulate—particularly NF-κB, MAPK, JAK-STAT, and TGF-β/Smad cascades (Fig. 2). For each virus, we traced the downstream consequences of miRNA regulation to the specific immune cells affected, revealing how miRNA networks ultimately shape macrophage function, T- and NK-cell activity, and antigen presentation.

Across these infections, a subset of shared miRNAs —notably miR-21, miR-146a, miR-150, and miR-155— emerges as a conserved regulatory core (Supplementary Table 5). These miRNAs fine-tune cytokine production, immune-cell differentiation, and antiviral responses by targeting overlapping sets of transcriptional regulators. Although core immune pathways repeatedly emerge as shared regulatory hubs targeted by conserved miRNAs, the downstream cellular effects differ by context: T-cell regulation predominates in HIV, antigen processing and NK-toxicity in HBV, NK-cell modulation in HSV and type I IFN response in SARS-CoV-2. These parallels suggest that diverse viruses converge on a limited set of signalling nodes but exploit them through distinct cellular routes, providing a unifying framework for understanding miRNA-driven immune control across viral infections.

Mechanistic examples summarised in Table 1 illustrate this diversity, while Supplementary Table 5 provides a comparative overview of miRNAs implicated across multiple infections, highlighting shared regulators and virus-specific adaptations.

**Table 1:** miRNA-mediated modulation of cellular signalling pathways in viral infections.

	miRNA	Target Gene	Effects	Ref.
**SARS-CoV-2**	miR-145, miR-146a, miR-155	JAK/STAT	MSC-EVs enhanced the expression of interferon-stimulated genes (ISGs), activating antiviral JAK/STAT signalling	[35]
	miR-21-5p, miR-22-3p	antiviral response genes	discrimination severe from mild/moderate Covid-19	[33]
	miR-155-5p, miR-224-5p	pro-inflammatory factors		
	miR-146a-5p	IFI44	IFN signalling pathways and regulation of immune responses	[24]
	miR-146a-5p,	IRAK1, TRAF6, CXCR4	opposite functions in inflammation regulation (i.e. IL-8 expression)	[23]
	miR-146a-3p	*DDX3X, RNF125 CXCR4*		
	miR-146a-5p, miR-23b-5p, let-7b-5p, miR-342-5p	Not reported	M2-EVs treatment reduce proinflammatory cytokine production such as TNF-α and IL-6; inhibited proinflammatory pathways (NF-κB, JAK-STAT and p38-MAPK)	[34]
	miR-150-5p	STAT1	modulating IFN-γ signalling pathway	[26]
	miR-155-3p	CHEK1 and CEP350, ERBB3	IFN response and cytokine signalling	[19]
	miR-155	RORγT, STAT3, FoxP3, SOCS1	Th17/Treg balance	[21]
	miR-155-5p	SOCS1	TLR activation and systemic inflammation	[20]
**Hepatitis B**	HBV-miR-3	SOCS5	activates the JAK/STAT signalling, promotes M1 polarisation	[51]
	miR-30e-5p	PRR-sensing and IFN signalling	promotes the expression of antiviral genes such as IFNβ, IFIT1, and IL6	[46]
	miR-128-3p	LINC01149 and MICA	induced NK cell-mediated cytotoxicity	[45]
	miR-212-3p	MAPK1	inhibits inflammatory cytokine production in macrophages	[52]
	miR-330-3p	CD244	mitigated apoptosis in CD8^+^ T-cells	[50]
**Human Immunodeficiency Virus**	miR-17-5p, miR-191-5p	nef, p21, SDF-1, XCL1, CCL2	inflammatory pathways, such as NF-κB, and influence immune responses.	[56]
	miR-155-5p miR-1248	Not reported	upregulated in early-treated individuals and correlated with HLA-DR and TIM-3 expression on CD56 NK cells	[58]
	miR-21-5p, miR-27b-3p, miR-146a-5p, miR-148-3p	Several targets	increased in HIV-positive subjects, correlated positively with metabolites associated with oxidative stress, may have protective anti-inflammatory effects during HIV pathogenesis.	[82]
	miR-21-5p, miR-155-5p	Not reported	upregulated in CSF-derived EVs from CT/HIV patients	[79]
	miR-27b, miR-29, miR-150, miR-221	Not reported	shows significant correlations with viral load, CD4^+^ T-cell count, and nef gene expression	[69]
	miR-28, miR-125b, miR-150, miR-155	Not reported	reduced after methadone treatment, whereas IFN-β addition reverted the inhibitory effect of methadone.	[85]
	miR-30 family	CD73 (NT5E)	inhibition of miRNAs (30b, 30c and 30e) resulted in significant upregulation of CD73 mRNA in CD8^+^ T cells	[66]
	miR-146a	IL-1β, TRAF6, IRAK1	Methamphetamine induces miR-146a and triggers an IL-1β auto-regulatory loop to modulate innate immune signalling in CD4^+^ T-cells	[86]
	miR-150	Not reported	decreased in immune cells; a potential biomarker to identify *Pneumocystis* pneumonia patients at high risk of death.	[73]
**Herpes Simplex Virus**	miR-21-5p, miR-146a-5p, miR-155-5p	Several targets	neuronal cell damage and oxidative stress associated exosomal miRNAs. Low expression levels of miR-138-5p correlated with low miR-H3-3p. miR-155-5p correlated with miR-H27 expression	[101]
	miR-27b, miR-199b, miR-369-3p, miR-491-3p	Several targets	upregulated in patients with HSV. Inhibition of them cause dysregulation of several genes associated with antiviral response involved in the TLR, NOD-like, RIG-I-like receptor and type I IFN signalling pathways.	[96]
	miR-29a-3p	TNF, IFN-γ, ACKR1	ACKR1 expression in endothelial cells enhances immune cell recruitment during the early phases of HEK	[111]
	miR-155	Not reported	upregulation indicates an active immune response and may be linked to oncogenesis	[103]

***CSF*** Cerebrospinal Fluid, ***EVs*** Extracellular Vesicles, ***HEK*** Herpes Epithelial Keratitis, **M2-EVs** M2 macrophage-derived extracellular vesicles, ***MSC–EVs*** Mesenchymal Stem cell-derived extracellular vesicles.

An underexplored yet crucial dimension of miRNA biology involves interactions between viral-encoded and host-derived miRNAs. Viral miRNAs may compete with host miRNAs for common binding sites, act as ceRNA-like sponges, or co-regulate immune-related targets, thereby fine-tuning infection outcomes. In HSV-1, multiple LAT-encoded miRNAs (e.g., miR-H2, miR-H3, miR-H4) regulate both viral and host transcripts to maintain latency; miR-H2 can antagonise host miR-155, reducing inflammation and supporting viral persistence [112, 113]. Similarly, in HBV, the viral HBV-miR-3 modulates both viral replication and host regulatory circuits, sustaining chronic infection [113, 114]. These host–virus miRNA interactions represent an additional regulatory layer that viruses exploit to balance immune evasion with long-term coexistence, underscoring the need for deeper experimental investigation.

An additional dimension of miRNA-mediated regulation is their cell-type specificity and temporal dynamics. The same miRNA can exert divergent effects depending on the immune cell subset and infection stage. For instance, miR-146a-5p dampens NF-κB signalling in macrophages, whereas miR-146a-3p promotes pro-inflammatory activity in other immune cells, while miR-150-5p shows early downregulation to enhance antiviral responses and later normalisation to support resolution of inflammation. Such spatiotemporal heterogeneity reflects differences in transcriptional control, cytokine feedback, and epigenetic regulation, underscoring the importance of considering timing and cellular context in therapeutic design. Nonetheless, clinical translation remains challenged by these issues of cell-type specificity, temporal expression dynamics, and interplay with other non-coding RNAs.

Circulating miRNAs are stable and measurable, offering promise as diagnostic and prognostic biomarkers that mirror immune activation, viral load, and treatment response. Strategies to inhibit pro-inflammatory miRNAs or deliver anti-inflammatory ones —including antagomirs, mimics, or EV-based delivery— could restore immune balance and limit pathology. In addition, viral miRNAs may themselves serve as therapeutic targets or vaccine adjuvants, given their roles in latency and immune modulation.

## Limitations and future perspectives

Despite considerable advances, this review is subject to several limitations that reflect the current state of the field. The breadth of available literature is uneven across viral infections; while SARS-CoV-2 and HIV have been extensively investigated in recent years, fewer studies address miRNA-mediated immune regulation in HBV and HSV, limiting the depth of comparative analysis. Moreover, methodological heterogeneity among studies, including differences in cell types, infection models, and normalisation strategies, hinders direct data integration. These disparities emphasise the need for standardised experimental and computational approaches to ensure reproducibility and comparability across viruses.

From a translational perspective, increasing evidence supports the potential of miRNAs as biomarkers, therapeutic targets, and vaccine adjuvants. Several clinical and preclinical studies are evaluating miRNA mimics or antagomirs in viral and inflammatory contexts (e.g., miR-122 inhibitors in hepatitis, miR-155 modulators in immune regulation). Nonetheless, challenges such as specific delivery, off-target interactions, and dynamic temporal regulation remain major barriers to clinical translation. Addressing these gaps through integrated multi-omics profiling, in vivo validation, and rational drug-delivery design will be crucial to transform mechanistic insights into tangible antiviral interventions.

## Supplementary information

Decoding miRNA-Mediated Immunoregulation in SARS-CoV-2, HBV, HIV, and HSV Infections Supplementary Tables
