# Supplementary material for: Decoding miRNA-Mediated Immunoregulation in SARS-CoV-2, HBV, HIV, and HSV Infections
Source: Genes Immun. 2026 Jan 20;27(1):1–12. doi: 10.1038/s41435-026-00376-4 (PMC12923359; doi:10.1038/s41435-026-00376-4)
Supplement: Supplementary file 1 — Decoding miRNA-Mediated Immunoregulation in SARS-CoV-2, HBV, HIV, and HSV Infections Supplementary Tables [file 41435_2026_376_MOESM1_ESM.docx]

**Decoding miRNA-Mediated Immunoregulation in
SARS-CoV-2, HBV, HIV, and HSV Infections**

**Supplementary Tables**

**Supplementary Table 1:** miRNAs and immunoregulatory roles in SARS-CoV-2 infections

| **miRNA** | **Sample/Cell** | **Target Gene** | **Effects** | **Ref.** |
| --- | --- | --- | --- | --- |
| miR-7,  miR-18a,  miR-29b,  miR-124,  miR-145,  miR-146a,  miR-155 | MSC–EVs and hamster model | JAK/STAT | MSC-EVs enhanced the expression of interferon-stimulated genes (ISGs), activating antiviral JAK/STAT signalling | [35] |
| miR-16-2-3p,  miR-618 | Serum | Not Reported | positively correlated with IL-1β expression and outcomes of SARS-Cov-2 infection | [27] |
| miR-18b-5p | HEK293 | NSP4 | mutations on miRNA binding sites reduce or abolish the binding ability | [16] |
| miR-197-5p |  | NSP3 |  |  |
| miR-20a-5p,  miR-29b-2-5p,  miR-142-5p,  miR-505-5p,  miR-6501-5p | Blood | TNFRSF1B, FCGR2A, STAT3, TLR2 | cytokine storm and immune dysregulation | [29] |
| miR-21-5p,  miR-22-3p | Serum samples, Vero E6 and Calu-3 cells | antiviral response genes | discrimination severe from mild/moderate Covid-19 | [33] |
| miR-155-5p, miR-224-5p |  | pro-inflammatory factors |  |  |
| miR-26a-5p  miR-30c-5p | EVs and plasma | CNR1 | regulates endocannabinoid signalling included VEGFA, GNAI2, IGF1, BDNF, IGF1R, CREB1 and CCND1 | [36] |
| miR-146a-5p |  | CNR2 (CB2) |  |  |
| miR-199a-5p miR-370-5p |  | MGLL |  |  |
| miR-27a/b,  miR-369-3p,  miR-491-5p | NK-92MI | SNARE  and ACE2 | pro-inflammatory cytokines, particularly TNF-α, reducing inflammation severity | [11] |
| miR-29 family | dataset  GSE157103 | ORF1ab | binds SARS-Cov-2 genome, targets differently expressed genes | [17] |
| miR-93-5p miR-150-5p | Blood | Not reported | immune cell functions | [32] |
| miR-98-5p | LUAD | TMPRSS2 | CD8^+^ and CD4^+^ T-cell infiltration | [5] |
| miR-148a-3p | LUSC | NRP1 | worse survival in LUSC |  |
| miR-136-3p,  miR-369-5p | HBE cells | ACE2 | TGF-β1 inhibits ACE2 by miR-136-3p and miR-369-5p; decreases entry of SARS-CoV-2 into the host cells, decreases anti-inflammatory and anti-fibrotic effects of ACE2 | [10] |
| miR-143-3p | Blood | BCL2 | affects neutrophil count and neutrophil-to-lymphocyte ratio through the expression of BCL2 | [28] |
| miR-144 | *Datasets | Not reported | regulates IgA nephropathy with ATF1 | [39] |
| miR-146a-5p  miR-1-3p | **RA datasets | IFI44 | interferon signalling pathways and regulation of immune responses | [24] |
| miR-146a-5p, | A549, HL-60, THP1 | IRAK1, TRAF6, CXCR4 | opposite functions in inflammation regulation  (i.e. IL-8 expression) | [23] |
| miR-146a-3p |  | DDX3X, RNF125 *CXCR4 (shared) |  |  |
| miR-146a-5p, miR-23b-5p,  let-7b-5p,  miR-342-5p | RAW264.7, mMφ, mouse model | Not reported | M2-EVs treatment reduce proinflammatory cytokine production such as TNF-α and IL-6; inhibited proinflammatory pathways (NF-κB, JAK-STAT and p38-MAPK) | [34] |
| miR-150-5p | PBMCs | STAT1 | modulating IFN-γ signalling pathway | [26] |
| miR-155-3p | lung epithelial  cells | CHEK1 and  CEP350, ERBB3 | interferon response and cytokine signalling | [19] |
| miR-483-3p |  | LGALS9, SETD5, KCNQ3, ARGHAP5, DAG1 |  |  |
| miR-155 | PBMCs | RORγT, STAT3, FoxP3, SOCS1 | Th17/Treg balance | [21] |
| miR-155-5p miR-200c-3p | Serum | Not Reported | discriminated between mild and moderate cases | [22] |
| miR-155-5p | Blood | SOCS1 | TLR activation and systemic inflammation | [20] |
| miR-208a-3p,  miR-323-3p | Serum | ACE2  positive circulating exosomes | downregulated in ExoACE2 | [12] |
| let-7g-5p;  miR-4454+  miR-7975 |  |  | upregulated in ExoACE2 |  |
| miR-298 | nasopharyngeal swabs, GSE148729 | circ_3205 | regulates KCNMB4 and PRKCE expression, relates with immune response and blood coagulation | [30] |
| miR-369-3p | Serum | several predicted targets | regulation of immune pathways by targeting genes in the T-Cell Receptor and co-stimulatory signalling; IL-4, Notch, and TGF-beta signalling pathways | [25] |
| miR-1207-5p | Data sets | CSF1 | targets spike sequence, deregulates CSF-1 to enhance inflammatory responses, upregulates EMT-related genes | [18] |
| miR-1246 | Calu-3, Vero E6 | ACE2 | involved in RAF/MEK/ERK, PI3K/AKT, and JAK/STAT pathways linked to inflammation and cellular stress. | [9] |
| miR-4324 | A549, H460 | LINC01002,  FRMD8 | inhibition of the host's antiviral immune response | [31] |
| SCV2-miR-OR1ab-1-3p, SCV2-miR-OR1ab-2-5p | Data sets, HEK293T | STAT1 and STAT2 | inhibit host type I interferon response | [13] |
| miR-O7a.1/2 | ACE2‐expressing A549 | BATF2 | target interferon‐stimulated genes and represses their expression | [14] |

***EVs:*** *Extracellular Vesicles,* ***ExoACE2****: Angiotensin-Converting Enzyme 2 Expressing Exosome,* ***HBE:*** *Human Bronchial Epithelial,* ***ICU****:* *Intensive Care Unit,* ***LUAD****:* *Lung Adenocarcinoma,* ***LUSC****:* *Lung Squamous Cell Carcinoma,* ***M2-EVs:*** *M2 macrophage-derived extracellular vesicles,* ***mMφ:*** *Primary mouse peritoneal macrophages,* ***MSC–EVs:*** *Mesenchymal Stem cell-derived* *extracellular vesicles,* ***NHBE****:* *SARS-Cov-2-Infected Human Bronchial Epithelial,
*Datasets: GSE201535 and GSE104948**********Rheumatoid arthritis (RA) datasets: GSE93272, GSE33341, GSE17755, GSE55235, GSE13670.*

**Supplementary Table 2:** miRNAs and their effects on hepatitis B and immune response

| **miRNA** | **Cell** | **Target Gene** | **Effects** | **Ref.** |
| --- | --- | --- | --- | --- |
| HBV-miR-3 | THP-1,  HepG2-NTCP | SOCS5 | activates the JAK/STAT signalling promotes M1 macrophage polarization | [51] |
| miR‐7‐1‐1‐3p, miR‐192‐5p, miR‐195‐5p, miR‐32‐5p | Dendritic cells from PBMCs | CANX, CALR, HSPA1B, HSPA8, HSP90AB1,  HLA‐A and HLA‐B | upregulated in low replicative group | [44] |
| miR‐342‐3p, miR‐940 |  |  | upregulated in HBe negative group |  |
| miR‐34a‐5p, miR‐130b‐3p, miR‐221‐3p, miR‐320a, miR‐324‐5p, miR‐484 |  |  | upregulated in acute viral hepatitis group |  |
| miR‐2278, miR‐615‐3p, miR‐3681‐3p |  |  | downregulated in immune active group compared healthy control |  |
| miR‐152‐3p, miR‐3613‐3p |  |  | downregulated in low replicative group vs immune active |  |
| miR‐152‐3p, miR‐503‐3p |  |  | downregulated in HBe negative group compared low replicatives |  |
| miR-20a-5p | PBMCs, THP-1  LX-2 | Not reported | modulating macrophage polarization and hepatic stellate cell activation | [47] |
| miR-340-5p |  |  |  |  |
| miR-30e-5p | Blood samples and cell lines | negative regulators of PRR-sensing and IFN signalling pathways | promotes the expression of antiviral genes such as IFNβ, IFIT1, and IL6 | [46] |
| miRNA-93 | Datasets (GSE67882, GSE69580), HepG2 | GRB2 | modulates immune cell infiltration in HCC, particularly affecting CD4^+^ T-cells, macrophages and neutrophils | [49] |
| miR-99a-5p, miR-122-5p, miR-192-5p | Serum | Not reported | response to ALT levels, reflecting their sensitivity to liver damage and inflammation | [41] |
| miR-128-3p | HepG2 | LINC01149 and MICA | induced NK cell-mediated cytotoxicity | [45] |
| miR-138-5p | HepaRG | APOBEC3B (A3B) | post-transcriptionally downregulates APOBEC3B (NF-κB target) | [54] |
| miR-212-3p | RAW264.7, THP1, U937, PBMs | MAPK1 | inhibits HBeAg-induced inflammatory cytokine production in macrophages | [52] |
| miR-223-3p,  miR-483-3p | Serum | Several  predicted targets | regulation of inflammation, immune response, and tumorigenesis | [42] |
| miR-330-3p | PBMCs and cell lines | CD244 | mitigated apoptosis in CD8^+^ T-cells | [50] |
| miR-433-3p,  miR-378 family | HSCs | ASPM | immune cell infiltration | [43] |
| miR-939 | Monocytes from PBMCs and  cell lines | Not reported | activates the MAPK-p38-IL-8 signalling; boosts IL-8 production | [53] |
| miR-6840-3p | PBMCs,  HEK293T | JADE2 | inflammatory signalling | [48] |
| miR-6861-3p |  | NR3C2 | reduced in patients with poor prognosis |  |
| amiRNA-135 | HEK-293T, HepG2, mice model | HBV genome | suppressed HBV replication and antigen production | [55] |

***ALT****: Alanine Transaminase,* ***ASPM****: Abnormal Spindle-Like Microcephaly-Associated Gene,* ***ECM****: Extracellular Matrix,* ***HBV****: Hepatitis B Virus,* ***HSCs****: Hematopoietic Stem Cells,* ***HepaRG****: Non-Transformed Progenitor Cell Line that can be Differentiated into Hepatocytes,* ***MHC****: Major Histocompatibility Complex,* ***PBMs:*** *Peripheral Blood Monocytes*

**Supplementary Table 3:** miRNAs involved in immune regulation during HIV-1 infection

| miRNA | Sample/Cell | Target Gene | Effects | Ref. |
| --- | --- | --- | --- | --- |
| let-7d-5p | Plasma | Not reported | a potential biomarker, the most highly differentially expressed miRNA, downregulated in the immunological nonresponders | [72] |
| miR-7 | Rat primary astrocytes, PRHNs, A172 | NLGN2 | downregulation of NLGN2 by miR-7 causes synaptic injury, affecting both inhibitory (GAD65) and excitatory (vGlut1) synapses | [80] |
| miR-10a-5p | Primary T-cells | TFRC, SDC1, and  ACTG1 | inhibition of miR-10a-5p enhance CD8^+^ T-cell cytotoxicity *ex vivo* in patients with chronic HIV-1 infection on long-term ART. | [65] |
| miR-15a,  miR-24 | Primary T-cells | Not reported | secreted from HIV-1-infected CD4^+^ T-cells; inhibited CD34^+^ haematopoietic progenitor stem cell differentiation into myeloid and erythroid colonies | [68] |
| miR-16-5p, miR-138-5p, miR-323-3p, miR-580, miR-627 | Plasma | Not reported | negatively correlated with both CD4^+^ T-cell counts and the increase in the proportion of CD4^+^ T cells | [67] |
| miR-17-5p, miR-191-5p | PBMCs | nef, p21, SDF-1,  XCL1, CCL2 | inflammatory pathways, such as NF-κB,  and influence immune responses. | [56] |
| miR-106a-5p,  miR-125a,   miR140-3p, miR-339-3p |  | Not reported | increased in comparison to the HIV/HBV- as well as HIV/HCV-infected people, discriminating ECs from ART-naïve HIV-infected groups. |  |
| miR-18a-3p, miR-296-5p, miR-501-3p, miR-548d-5p | PBMCs | Not reported | downregulated in EARLY treated group of patients | [58] |
| miR-155-5p  miR-1248 |  |  | upregulated in early-treated individuals and correlated with HLA-DR and TIM-3 expression on CD56 NK cells |  |
| miR-21  miR-146, miR-155 | PBMCs, | Not reported | positive correlation with IL-10 and CD19 | [90] |
| miR-21  let-7a-5p,  let-7c-5p | SIV model,  mice primary astrocytes | Not reported | THC treatment induces neuro protective/ anti-inflammatory factors that mediated by EV-associated miRNAs. | [81] |
| miR-21-5p, miR-27b-3p, miR-146a-5p, miR-148-3p,  miR-423-5p, | Plasma EVs | several targets in oxidative stress response; IFN-Υ, TGF-β, Notch, TLR signalling; T- cell activation | increased in HIV-positive subjects, correlated positively with metabolites associated with oxidative stress, may have protective anti-inflammatory effects during HIV pathogenesis. | [82] |
| miRNA-21-5p, miRNA-23a-3p, miRNA-24-2-5p, miR-124-3p | Serum | Not reported | significantly altered in HIV+ osteopenia+ individuals; biomarkers in distinguishing HIV+ individuals with osteoporosis. | [70] |
| miR-21-5p  miR-155-5p | Serum and  CSF samples | Not reported | upregulated in CSF-derived EVs from CT/HIV patients | [79] |
| miR-23a-3p,  miR-4685-5p,  miR-425-5p,  miR-532-5p,  miR-1288 | PBMCs | Several predicted targets | miRNA–mRNA networks in monitoring immune recovery and disease trajectory | [78] |
| miR-26a | PBMCs derived CD4^+^ T-cells, HEK-293T, CEM.NKR-CCR5 cells | CD59 | promote ADCML of HIV-1 by reducing CD59 packaging into released virions | [84] |
| miR-27b,  miR-29, miR-150,  miR-221 | PBMCs | Not reported | shows significant correlations with viral load, CD4^+^ T-cell count, and nef gene expression | [69] |
| miR-28,  miR-125b, miR-150, miR-155 | BMDM | Not reported | reduced after methadone treatment, whereas IFN-β addition reverted the inhibitory effect of methadone. | [85] |
| miR-29a-3p | Blood | Not reported | negative correlation with HIV load,  modulates host immune response to HIV and similar viral infections | [61] |
| miR-30 family | PBMCs, CD8^+^ T | CD73 (NT5E) | inhibition of miRNAs (30b, 30c and 30e) resulted in significant upregulation of CD73 mRNA in CD8^+^ T cells | [66] |
| miR-33b-5p | Serum samples and HIV-1 infected  cell lines | ABCA1 | target *ABCA1* and caused aberrant lipid transport | [93] |
| miR-99b-5p, miR-200c-3p, miR-let-7i, miR-3120-3p | Blood | Several predicted targets | Positively correlated with motor, executive function, attention, language | [76] |
| miR-101, miR-145, miR-302 | Serum | PRKACB, RANKL  and SMAD3 | zoledronic acid treatment attenuated RANKL‑induced activation of NF‑κB and JNK signalling pathways | [87] |
| miR-103 | CD4^+^ T,  HEK-293T | CCR5 | involved in the transition of CD4^+^ T-cells from activated to memory state, prone to HIV-1 latent infection | [59] |
| miR-122-5p | Plasma | VEGF | serves as a biomarker linked to VEGF signalling and angiogenesis, potentially affecting immune responses in both AHI and CHI stages | [71] |
| miR-124-3p miR-144-3p, miR-144-5p, miR-183-5p, miR-451a, miR-4732-3p | PBMCS | Not reported | Dysregulated miRNAs in during atherosclerosis in HIV positive persons and associated with pro-inflammatory cytokines | [75] |
| miR-125b | HEK-293T, SupT1, T-lymphoblast and THP1 | CPSF6 | activation of miR-125b–mediated regulation of CPSF6 to promote HIV-1 nuclear entry | [62] |
| miR-128-3p | Blood | Several predicted targets | regulating cell cycle progression, apoptosis, immune responses, and cancer development | [83] |
| miR-139-5p | SIV model, PBMCs, CD14^+^  monocytes | PDE4D | modulates immune responses and plays crucial roles in vaccine efficacy | [92] |
| miR-146a | CD4^+^ T | IL-1β, TRAF6,  IRAK1 | Methamphetamine induces miR-146a and triggers an IL-1β auto-regulatory loop to modulate innate immune signalling in CD4^+^ T-cells | [86] |
| miR-150 | Mice model, Clinical samples  T-, B-cells and macrophages | Not reported | decreased in immune cells; a potential biomarker to identify *Pneumocystis* pneumonia patients at high risk of death. | [73] |
| miR-191-5p | PBMCs | NUP50, CCR1 | acts as an inhibitor of HIV-1 infection by regulating NUP50 expression | [60] |
| miR-192-5p | PBMCs | Several predicted targets involved in inflammatory pathways | down regulated in patients exhibiting ocular manifestations maintains; distinguish between HIV patients diagnosed with Immune Recovery Uveitis. | [77] |
| miR-543 |  |  |  |  |
| miR-210-5p | Serum samples, HOS and MT4 cells | TGIF2 | upregulated by HIV-1 Vpr protein to induce G2 arrest by targeting TGIF2 | [63] |
| miR-223-3p  miR-1183  miR-8063 | Monocyte-depleted PBMCs | Not reported | significantly down-regulated in dendritic cell-based therapeutic vaccination responders | [91] |
| miR-320a-3p | Datasets, clinical samples and cell lines | FKBP5 | modulates FKBP5 expression in HIV/TB co-infection and may influence immune responses | [74] |
| miR-505 | mPMS, human brain tissues | SIRT3 | up regulated by HIV TAT protein; significant role in microglial senescence and neuroinflammation | [64] |
| miR-1246 | EVs from HIV infected, Morphine treated PBMCs, SHSY5Y cells | Not reported | up-regulated in the presence of morphine, morphine exposure could directly modulate the exosomes released by peripheral immune cells | [88] |
| miR-1297 | Clinical samples, CCC-HIE-2 cell | PLCβ1 | overexpression downregulated the tight junction protein ZO-1, which led to the blocked cytoskeleton rearrangement during epithelial cell repair, decreased activity, and increased permeability of the model cells, finally leading to cell apoptosis. | [94] |
| miR-4726-5p | Mice model, Primary Astrocytes | LINC01133, NDUFA9,  LIPG, KYNU, HKDC1 | HIV-1 Tat- and cocaine-mediated astrocyte dysfunction | [89] |

***ADCML:*** *antibody-dependent complement-mediated lysis****, AHI****: Acute HIV Infections,* ***BMDM:*** *Blood Monocyte-Derived Macrophages,* ***CHI****: Chronic HIV* *Infections,* ***CSF****: Cerebrospinal Fluid,* ***mPMs****: Mouse Primary Microglial* *Cells,* ***PRHNs****: Primary Rat Hippocampal Neurons,* ***SIV****: Simian Immunodeficiency Virus,* ***THC:*** *delta-9-tetrahydrocannabinol*

**Supplementary Table 4:** miRNAs and immunoregulatory roles in herpes simplex infections

| miRNA | Cell | Target Gene | Effects | Ref. |
| --- | --- | --- | --- | --- |
| miR-H1,  miR-H6 | HEK-293T, Rabbit skin and mouse neuroblastoma cells | UL30, LAT | regulate HSV-1 reactivation | [98] |
| miR-H2 | HEK293, human and mouse trigeminal ganglia cells | ICP0, ICP4,  FIH-1 | hyper-editing (A-to-G substitution) of miR-H2 by ADAR enzyme in neurons increases its target range | [100] |
| miR-H2 | HEK293T and  LX-2 | SMAD3 and SMAD4 | LAT-derived miRNAs negatively regulate TGF-β/Smad signalling and the expression of pro-fibrotic markers through targeting *SMAD3* and *SMAD4.* | [95] |
| miR-H3 |  | SMAD4 |  |  |
| miR-H4 |  | SMAD3 |  |  |
| miR-H2-3p | CSF-derived  exosomes | Several targets | positively detected miRNA species in exosomal preparations. miR-H27 associated with inflammatory responses and increased NfL levels. | [101] |
| miR-H3-3p |  |  |  |  |
| miR-H4-3p/5p |  |  |  |  |
| miR-H6-3p |  |  |  |  |
| miR-H27 |  |  |  |  |
| miR-21-5p |  |  | neuronal cell damage and oxidative stress associated exosomal miRNAs. Low expression levels of miR-138-5p correlated with low expression levels of miR-H3-3p.  miR-155-5p correlated with miR-H27 expression |  |
| miR-146a-5p |  |  |  |  |
| miR-138-5p |  |  |  |  |
| miR-155-5p |  |  |  |  |
| miR-H6 | Subgingival tissue samples | ICP4 | contributed to periodontal inflammation and disease progression | [106] |
| miR-H8 | Rabbit skin cells | GPI-anchor biosynthesis pathway | miR-H8 is not essential for HSV-1 replication in skin epithelial cells | [97] |
|  | Neuro2A (mouse neuroblastoma) |  | miR-H8 strongly supports HSV-1 replication in mouse neuronal cells during acute infection. |  |
|  | LUHMES |  | miR-H8 may suppress replication in human post-mitotic neurons, possibly to promote latency or limit cytopathic effects |  |
| miR-H16 | Orthotopic glioma- mouse models | FIH-1 | FIH-1 downregulation enhances NOTCH ligand ubiquitination and signalling in glioma cells | [99] |
| miR-27b | PBMCs,  NK‑92 cells | Several targets | upregulated miRNAs in patients with HSV infection. Selective inhibition of them resulted in down- or upregulation of several genes associated with antiviral response involved in the modulation TLR, NOD-like receptor, RIG-I-like receptor and type I IFN signalling pathways. | [96] |
| miR-199b |  |  |  |  |
| miR-369-3p |  |  |  |  |
| miR-491-3p |  |  |  |  |
| miR-29a-3p | Tear samples | TNF, IFN-γ,  ACKR1 | ACKR1 expression in endothelial cells enhances immune cell recruitment during the early phases of HEK | [111] |
| miR-96,  miR-182,  miR-183 | Cell lines | FoxO family | PI3K/Akt pathway and innate immune responses are subject to modulation | [108] |
| miR-155 | GaHV-2-infected CEF | Not reported | miR-155 upregulation indicates an active immune response and may be linked to oncogenesis | [103] |
| miR-181b-5p  miR-222-3p  miR-338-3p  miR-635 | HCECs | Not reported | regulate antiviral and inflammatory responses via circRNA interaction in HSV-1–infected corneal cells | [107] |
| kshv-miR- K12-1-5p | human heart and plasma samples,  C57BL/6J mice model, cell lines | OAS1, OAS2, OAS3, MX1, IFIT1, IFIT3, IRF7, and RSAD2 | antiviral response, enhancing viral infection, and promoting cardiac inflammation | [104] |

***CEF****: Chicken Embryo Fibroblasts,* ***CSF****: Cerebrospinal Fluid,* ***HCECs****: Human Corneal Epithelial Cells,* ***HCMECs****: Human Cardiac Microvascular Endothelial Cells,* ***HEK****: Herpes Epithelial Keratitis* ***LUHMES:*** *Lund human mesencephalic cells****,*** ***GaHV-2:*** *Gallid Herpes Virus 2,* ***ECM:*** *Extracellular Matrix*

**Supplementary Table 5:** Comparative overview of miRNAs implicated in multiple viral infections

| **miRNA** | **Reported infections** | **Target Gene** | **Effects** | **Ref.** |
| --- | --- | --- | --- | --- |
| miR-20a-5p | SARS-CoV-2 | TNFRSF1B, FCGR2A, STAT3, TLR2 | cytokine storm and immune dysregulation | [29] |
|  | HBV | Not reported | modulating macrophage polarization and hepatic stellate cell activation | [47] |
| miR-21-5p | SARS-CoV-2 | antiviral response genes | discrimination severe from mild/moderate Covid-19 | [33] |
|  | HIV | several targets in oxidative stress response; IFN-Υ, TGF-β, Notch, TLR signalling; T- cell activation | increased in HIV-positive subjects, correlated positively with metabolites associated with oxidative stress, may have protective anti-inflammatory effects during HIV pathogenesis. | [82] |
|  |  | Not reported | upregulated in CSF-derived EVs from CT/HIV patients | [79] |
|  | HSV | Several targets | neuronal cell damage and oxidative stress associated exosomal miRNAs. | [101] |
| miR-27b | HIV | Not reported | shows significant correlations with viral load, CD4^+^ T-cell count, and nef gene expression | [69] |
|  | HSV | Several targets | upregulated miRNAs in patients with HSV infection. Selective inhibition of them resulted in down- or upregulation of several genes associated with antiviral response involved in the modulation TLR, NOD-like receptor, RIG-I-like receptor and type I IFN signalling pathways. | [96] |
| miR-29a-3p | HIV | Not reported | negative correlation with HIV load,  modulates host immune response to HIV and similar viral infections | [61] |
|  | HSV | TNF, IFN-γ,  ACKR1 | ACKR1 expression in endothelial cells enhances immune cell recruitment during the early phases of HEK | [111] |
| miR-122-5p | HBV | Not reported | response to ALT levels, reflecting their sensitivity to liver damage and inflammation | [41] |
|  | HIV | VEGF | serves as a biomarker linked to VEGF signalling and angiogenesis, potentially affecting immune responses in both AHI and CHI stages | [71] |
| miR-128-3p | HBV | LINC01149 and MICA | induced NK cell-mediated cytotoxicity | [45] |
|  | HIV | Several predicted targets | regulating cell cycle progression, apoptosis, immune responses, and cancer development | [83] |
| miR-138-5p | HBV | APOBEC3B (A3B) | post-transcriptionally downregulates APOBEC3B (NF-κB target) | [54] |
|  | HIV | Not reported | negatively correlated with both CD4^+^ T-cell counts and the increase in the proportion of CD4^+^ T cells | [67] |
|  | HSV | Several targets | neuronal cell damage and oxidative stress associated exosomal miRNAs. Low expression levels of miR-138-5p correlated with low expression levels of miR-H3-3p | [101] |
| miR-145 | SARS-CoV-2 | JAK/STAT | MSC-EVs enhanced the expression of interferon-stimulated genes (ISGs), activating antiviral JAK/STAT signalling | [35] |
|  | HIV | PRKACB, RANKL  and SMAD3 | zoledronic acid treatment attenuated RANKL‑induced activation of NF‑κB and JNK signalling pathways | [87] |
| miR-146a | SARS-CoV-2 | JAK/STAT | MSC-EVs enhanced the expression of interferon-stimulated genes (ISGs), activating antiviral JAK/STAT signalling | [35] |
|  | HIV | IL-1β, TRAF6,  IRAK1 | Methamphetamine induces miR-146a and triggers an IL-1β auto-regulatory loop to modulate innate immune signalling in CD4^+^ T-cells | [86] |
| miR-146a-5p | SARS-CoV-2 | IFI44 | interferon signalling pathways and regulation of immune responses | [24] |
|  |  | CNR2 (CB2) | regulates endocannabinoid signalling included VEGFA, GNAI2, IGF1, BDNF, IGF1R, CREB1 and CCND1 | [36] |
|  |  | IRAK1, TRAF6, CXCR4 | opposite functions in inflammation regulation  (i.e. IL-8 expression) | [23] |
|  |  | Not reported | M2-EVs treatment reduce proinflammatory cytokine production such as TNF-α and IL-6; inhibited proinflammatory pathways (NF-κB, JAK-STAT and p38-MAPK) | [34] |
|  | HIV | several targets in oxidative stress response; IFN-Υ, TGF-β, Notch, TLR signalling; T- cell activation | increased in HIV-positive subjects, correlated positively with metabolites associated with oxidative stress, may have protective anti-inflammatory effects during HIV pathogenesis. | [82] |
|  | HSV | Several targets | neuronal cell damage and oxidative stress associated exosomal miRNAs. | [101] |
| miR-155 | SARS-CoV-2 | JAK/STAT | MSC-EVs enhanced the expression of interferon-stimulated genes (ISGs), activating antiviral JAK/STAT signalling | [35] |
|  |  | RORγT, STAT3, FoxP3, SOCS1 | Th17/Treg balance | [21] |
|  | HIV | Not reported | positive correlation with IL-10 and CD19 | [90] |
|  |  |  | reduced after methadone treatment, whereas IFN-β addition reverted the inhibitory effect of methadone. | [85] |
|  | HSV |  | miR-155 upregulation indicates an active immune response and may be linked to oncogenesis | [103] |
| miR-155-5p | SARS-CoV-2 | pro-inflammatory factors | discrimination severe from mild/moderate Covid-19 | [33] |
|  |  | Not Reported | discriminated between mild and moderate cases | [22] |
|  |  | SOCS1 | TLR activation and systemic inflammation | [20] |
|  | HIV | Not reported | Upregulated in early-treated individuals and correlated with HLA-DR and TIM-3 expression on CD56 NK cells | [58] |
|  |  |  | upregulated in CSF-derived EVs from CT/HIV patients | [79] |
|  | HSV | Several targets | miR-155-5p correlated with miR-H27 expression | [101] |
| miR-192-5p | HBV | Not reported | response to ALT levels, reflecting their sensitivity to liver damage and inflammation | [41] |
|  | HIV | Several predicted targets involved in inflammatory pathways | down regulated in patients exhibiting ocular manifestations maintains; distinguish between HIV patients diagnosed with Immune Recovery Uveitis. | [77] |
| miR-323-3p | SARS-CoV-2 | ACE2 positive circulating exosomes | downregulated in ExoACE2 | [12] |
|  | HIV | Not reported | negatively correlated with both CD4^+^ T-cell counts and the increase in the proportion of CD4^+^ T cells | [67] |
| miR-369-3p | SARS-CoV-2 | SNARE  and ACE2 | pro-inflammatory cytokines, particularly TNF-α, reducing inflammation severity | [11] |
|  |  | several predicted targets | regulation of immune pathways by targeting genes in the T-Cell Receptor and co-stimulatory signalling; IL-4, Notch, and TGF-beta signalling pathways | [25] |
|  | HSV | Several targets | upregulated miRNAs in patients with HSV infection. Selective inhibition of them resulted in down- or upregulation of several genes associated with antiviral response involved in the modulation TLR, NOD-like receptor, RIG-I-like receptor and type I IFN signalling pathways. | [96] |
| miR-1246 | SARS-CoV-2 | ACE2 | involved in RAF/MEK/ERK, PI3K/AKT, and JAK/STAT pathways linked to inflammation and cellular stress. | [9] |
|  | HIV | Not reported | up-regulated in the presence of morphine, morphine exposure could directly modulate the exosomes released by peripheral immune cells | [88] |
